# Beta-Lactam Antibiotics Can Be Measured in the Exhaled Breath Condensate in Mechanically Ventilated Patients: A Pilot Study

**DOI:** 10.3390/jpm13071146

**Published:** 2023-07-17

**Authors:** José Escalona, Dagoberto Soto, Vanessa Oviedo, Elizabeth Rivas, Nicolás Severino, Eduardo Kattan, Max Andresen, Sebastián Bravo, Roque Basoalto, María Consuelo Bachmann, Kwok-Yin Wong, Nicolás Pavez, Alejandro Bruhn, Guillermo Bugedo, Jaime Retamal

**Affiliations:** 1 Departamento de Medicina Intensiva, Facultad de Medicina, Pontificia Universidad Católica de Chile, Santiago 8331150, Chile; josan553@gmail.com (J.E.); d1s4t4@gmail.com (D.S.); va403028@gmail.com (V.O.); elizabeth.rivasg1989@gmail.com (E.R.); nseverin@med.puc.cl (N.S.); e.kattan@gmail.com (E.K.); andresen@med.puc.cl (M.A.); sbravo@ucchristus.c (S.B.); roque.basoalto@gmail.com (R.B.); mcbachmann@uc.cl (M.C.B.); nzevap@gmail.com (N.P.); alejandrobruhn@gmail.com (A.B.); gbugedo@gmail.com (G.B.); 2 Unidad de Paciente Crítico, Hospital El Salvador, Santiago 8331150, Chile; 3 Programa de Farmacología y Toxicología, Facultad de Medicina, Pontificia Universidad Católica de Chile, Santiago 8331150, Chile; 4 Programa de Medicina Física y Rehabilitación, Red Salud UC-CHRISTUS, Santiago 8331150, Chile; 5 State Key Laboratory of Chemical Biology and Drug Discovery, Department of Applied Biology and Chemical Technology, Hong Kong Polytechnic University, Kowloon 999077, Hong Kong; kwok-yin.wong@polyu.edu.hk; 6 Departamento de Medicina Interna, Facultad de Medicina, Universidad de Concepción, Concepción 4030000, Chile

**Keywords:** exhaled breath condensate, antibiotics, pneumonia, heat and moisture exchange filter, HMEF, mechanical ventilation

## Abstract

Different techniques have been proposed to measure antibiotic levels within the lung parenchyma; however, their use is limited because they are invasive and associated with adverse effects. We explore whether beta-lactam antibiotics could be measured in exhaled breath condensate collected from heat and moisture exchange filters (HMEFs) and correlated with the concentration of antibiotics measured from bronchoalveolar lavage (BAL). We designed an observational study in patients undergoing mechanical ventilation, which required a BAL to confirm or discard the diagnosis of pneumonia. We measured and correlated the concentration of beta-lactam antibiotics in plasma, epithelial lining fluid (ELF), and exhaled breath condensate collected from HMEFs. We studied 12 patients, and we detected the presence of antibiotics in plasma, ELF, and HMEFs from every patient studied. The concentrations of antibiotics were very heterogeneous over the population studied. The mean antibiotic concentration was 293.5 (715) ng/mL in plasma, 12.3 (31) ng/mL in ELF, and 0.5 (0.9) ng/mL in HMEF. We found no significant correlation between the concentration of antibiotics in plasma and ELF (R^2^ = 0.02, *p* = 0.64), between plasma and HMEF (R^2^ = 0.02, *p* = 0.63), or between ELF and HMEF (R^2^ = 0.02, *p* = 0.66). We conclude that beta-lactam antibiotics can be detected and measured from the exhaled breath condensate accumulated in the HMEF from mechanically ventilated patients. However, no correlations were observed between the antibiotic concentrations in HMEF with either plasma or ELF.

## 1. Introduction

Pneumonia is a frequently encountered complication in critically ill patients and is known to be linked with significant morbidity and mortality rates [1]. The timely administration of appropriate antibiotic therapy has been demonstrated to reduce mortality associated with pneumonia in critically ill patients [2,3]. Furthermore, achieving therapeutic concentrations of antibiotics and ensuring their penetration into the infected tissues are crucial for effective infection control with antimicrobial treatment [4].

While measuring antibiotic plasma levels is a common practice in the ICU, it may not accurately reflect the actual concentration of antibiotics in the lung parenchyma. Following the intravenous administration of a beta-lactam antibiotic, the plasma concentration undergoes a characteristic rise and subsequent exponential decline. However, it is important to note that the peripheral compartments, such as the cellular and interstitial compartments, may exhibit different rates of change in their drug concentrations. The equilibrium between these compartments does not occur instantaneously, leading to potential differences in the concentration-time curves of the compartments. Despite these differences, plasma levels remain the primary clinical tool for guiding clinicians in adjusting antibiotic dosages.

Various techniques have been proposed for assessing tissue concentrations, including pulmonary microdialysis [5], tissue biopsies [6], and bronchoalveolar lavages (BALs) [7]. However, it is important to acknowledge that these techniques are invasive and can be associated with potentially adverse events. Among these techniques, BAL remains one of the most reliable methods for directly measuring antibiotic concentrations in the epithelial lining fluid (ELF) of the lungs. It provides valuable insights into the local pharmacokinetics of antibiotics and their distribution within lung tissues. However, it is essential to recognize that BALs are not without risks, particularly in critically ill patients. The invasive nature of BALs presents potential complications that limit their use, especially in specific patient populations. For example, patients with coagulopathy, such as those with bone marrow aplasia, may be at an increased risk of bleeding during the procedure. Additionally, there is a safety concern regarding the potential aerosolization of pathogens, which can pose a risk to both patients and healthcare providers, particularly in cases involving infectious agents like SARS-CoV-2 or tuberculosis [8,9].

An alternative non-invasive procedure for assessing lung conditions is the evaluation of exhaled breath condensate (EBC). This method has proven to be useful in various scenarios, including the categorization of acute respiratory distress syndrome (ARDS) severity [10] and the assessment of lung damage progression in experimental models [11]. McNeil et al. conducted a study in which they investigated whether exhaled breath condensate obtained from the heat and moisture exchange filter (HMEF) could serve as a representative sample of the distal airway. Through the proteomic analysis of the collected fluid, they were able to differentiate between cardiogenic acute pulmonary edema and ARDS, thereby distinguishing hydrostatic from inflammatory edema [12]. More recently, Herregots et al. demonstrated the identification of beta-lactam antibiotics in the exhaled air of spontaneously ventilating patients with pneumonia using high-performance liquid chromatography (HPLC) [13]. In preliminary studies conducted in our laboratory, we successfully detected antibiotics in the exhaled breath condensate collected from HMEFs [14]. By monitoring antibiotic concentrations in exhaled breath condensate, we aim to gain a deeper understanding of the pharmacokinetics of antibiotics in lung tissues, which is considered a critical factor in determining their efficacy in treating infections [13].

Therefore, the objective of the present study is to investigate whether the concentration of antibiotics measured in exhaled breath condensate collected from HMEF correlates with the concentration of antibiotics measured in the ELF from bronchoalveolar lavage (BAL) samples. This observational clinical study is designed to include mechanically ventilated immunosuppressed patients suspected of having pneumonia, for whom a BAL procedure is conducted to confirm or rule out the diagnosis of pneumonia. These patients are concurrently receiving beta-lactam antibiotics as part of their treatment regimen.

## 2. Materials and Methods

We conducted an observational clinical study in the Critical Care Unit of Hospital Clínico UC-Christus (Santiago, Chile) between January 2019 and January 2020. The study was designed in accordance with the guidelines of the Declaration of Helsinki, and it received approval from the Research Ethics Committee of the School of Medicine at Pontificia Universidad Católica de Chile (Approval No. 180925002/2019). Written informed consent was obtained from all patients to publish the findings of this study.

The study included consecutive patients who were connected to invasive mechanical ventilation and required a fiberoptic bronchoalveolar lavage (BAL) as indicated by the attending physician. Furthermore, these patients received beta-lactam antibiotics for a minimum duration of 6 h [15]. Exclusion criteria encompassed contraindications for using heat and moisture exchangers with filters (HMEFs), such as bronchopleural fistula, hemoptysis, excessive bronchorrhea, increased airway resistance, low-tidal-volume ventilatory strategy, and respiratory acidosis [16].

### 2.1. Patient Preparation

Once the patient was connected to mechanical ventilation, an HMEF (HME Filter 880530, EXXIMMED 2000, Shaoxing City, China) was installed into the ventilator circuit, specifically positioned between the Y-piece and the endotracheal tube. The HMEF utilized in our study possessed an internal volume of 49 mL and was composed of a cellulose-based heat and moisture exchanger (HME) in combination with an electrostatic polypropylene filter.

To ensure accurate and representative samples for analysis, we kept the HMEF in place for a minimum duration of 6 h before removing it from the ventilator circuit. This time interval was designed to allow sufficient time for the HMEF to capture and retain enough exhaled breath condensate and antibiotics within its structure for subsequent analysis.

### 2.2. Antibiotic Infusion

The selection of the appropriate beta-lactam antibiotic was at the discretion of the attending physician. The antibiotics were administered via an intravenous infusion according to previously published guidelines [17,18].

We included patients receiving ceftriaxone (Grifotriaxona, Laboratorio Chile, Santiago, Chile), piperacillin-tazobactam (Tratac, Laboratorio Chile, Santiago, Chile), imipenem (Imipenem, Fresenius Kabi, Santiago, Chile), or meropenem (Meronem, Pfizer, Santiago, Chile). According to the ICU protocol, the beta-lactam antibiotics were administered starting with a loading dose followed by prolonged infusions over 3–4 h, except for ceftriaxone which was administered as a daily bolus.

### 2.3. Bronchoalveolar Lavage

The bronchoscopic procedure was performed by an expert bronchoscopist. The tip of the bronchoscope was positioned in a subsegmental location, specifically targeting the area suspected of having a pneumonic process based on radiological findings. Bronchoalveolar lavage (BAL) was then carried out by sequentially instilling and recovering five separate 30 mL aliquots of 0.9% NaCl solution.

One of the BAL fluid (BALF) aliquots was promptly separated and transported to the laboratory. Upon arrival, the sample was centrifuged, allowing for the collection of the supernatant. This supernatant was then stored at a temperature of −80 °C until further analysis.

Considering that the introduction of the saline solution during the BAL procedure may result in a dilution effect on the BALF, we employed the urea concentration in both plasma and BALF to correct for this effect [19]. By utilizing urea concentration, we aimed to obtain a corrected value that would accurately reflect the concentration of the epithelial lining fluid (ELF). The measurement of urea concentrations in the BALF was performed using the UREA liquicolor Complete Test Kit (cat N 10505; Human Gesellschaft für Biochemica und Diagnostica mbH, Wiesbaden, Germany). 

### 2.4. Plasma Sampling

Simultaneously with the bronchoalveolar lavage (BAL), plasma samples were obtained from the patients. The samples were transported to the laboratory and centrifuged at 3500× *g* revolutions per minute (rpm) for 5 min, and then the supernatant was collected and stored at −80 °C until analysis.

### 2.5. HMEF Processing

During the preparation for BAL, HMEF was removed from the ventilatory circuit; both inspiratory and expiratory ports were carefully sealed to prevent the evaporation of the condensate and immediately transported to the laboratory using a refrigerated unit. 

To extract the condensate from the HMEF, the tube connection port of the HMEF was introduced into a sterile 50 mL falcon tube and securely fixed in place while ensuring the distal port remained sealed. Subsequently, the falcon tube plus the HMEF were centrifuged at 4500 rpm for 15 min at a temperature of 4 °C (Heraeus Megafuge™ 40r, Thermo Fisher Scientific, Langenselbold, Germany), allowing the separation of the condensate from the HMEF. The supernatant was collected and stored at −80 °C until analysis. In order to have a negative control for comparison, HMEFs soaked in 0.9% NaCl solution were used. These HMEFs served as a baseline to differentiate any potential contaminant present in the HMEF that could have been mistaken as beta-lactam antibiotics.

### 2.6. Analysis of Beta-Lactam Antibiotic Concentrations

To determine the concentrations of beta-lactam antibiotics in our study, we employed a fluorescent biosensor known as Pen-Pcf20. This biosensor is structurally related to beta-lactamase but lacks catalytic activity. When Pen-Pcf20 interacts with the antibiotic present in the medium, its intrinsic fluorescence undergoes changes over time, generating a characteristic pattern that allows for the identification of the specific antibiotic being analyzed. For each antibiotic, the fluorescence curves obtained by increasing antibiotic concentrations were analyzed. These curves were fitted to a 4-parameter sigmoid curve (4PL) with a variable dynamic range, which depends on the particular antibiotic being tested. Generally, the dynamic range spans from log −9 to log −3, with an inflection point typically around log −6. In cases where the fluorescence of a sample was very high or saturated (>log −6), the sample was successively diluted by a factor of 10 (titrated) until its fluorescence fell within the dynamic range of the technique. This dilution process allowed us to accurately determine the antibiotic concentration via extrapolation [20,21]. Samples whose estimated antibiotic concentration was less than the technique’s limit of detection (LOD), but which exhibited the pattern of fluorescence change over time characteristic of the expected antibiotic, were arbitrarily assigned the technique’s LOD value. This act was intended to prevent the misinterpretation of a low concentration as absent [22,23,24,25,26]. 

### 2.7. Statistical Analysis

Since there were no previous data regarding antibiotic levels in HMEF, and as this was an exploratory study, we did not perform a sample size calculation, and we arbitrarily decided to include 12 patients. The Shapiro–Wilk test was used to test data for normality. We expressed values as mean-standard deviation (SD) or median-range (IQR), where appropriate. We compared antibiotic levels between the three compartments analyzed (ELF, HMEF, and plasma) using a one-way (repeated measures) analysis of variance (ANOVA). *p* < 0.05 was considered statistically significant. We analyzed the correlation among the continuous variables using the Pearson correlation coefficient. The analysis was performed with GraphPad Prism version 8.00 for Mac (GraphPad Software, San Diego, CA, USA).

## 3. Results

In our analysis, we included a total of twelve patients, with the majority being female participants (*n* = 7). The mean age of the patients was 44 (20.9) years, and the mean body mass index (BMI) was 25.2 (6.9). All patients presented with respiratory failure upon admission to the intensive care unit and had immunosuppression as their primary diagnosis. Table 1 provides an overview of the main baseline patient characteristics. Nine patients received carbapenems, two patients were treated with piperacillin/tazobactam, and one patient received ceftriaxone.

**Figure 1:**
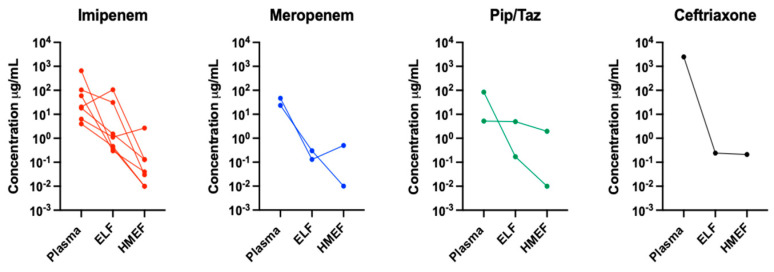
Concentration of the different antibiotics used in the study in the three compartments. ELF, epithelial lining fluid; HMEF, heat and moisture exchange filter.

During the study, an average volume of 50 (30) μL of exhaled breath condensate was collected from the HMEF. Antibiotics were detected in the plasma, exhaled breath condensate collected from HMEF, and BALF samples of every patient studied. In addition, we observed a decremental gradient of antibiotic concentrations from plasma to the epithelial lining fluid and further to the exhaled breath condensate from HMEF. However, no statistical difference in antibiotic concentrations was observed between ELF and exhaled breath condensate collected from the HMEF. The mean antibiotic concentration in plasma was 293.5 (715) ng/mL, 12.3 (31) ng/mL in ELF, and 0.5 (0.9) ng/mL in the HMEF exhaled breath condensate. Figure 1 illustrates the individual concentration of each antibiotic in each compartment examined.

**Figure 2:**
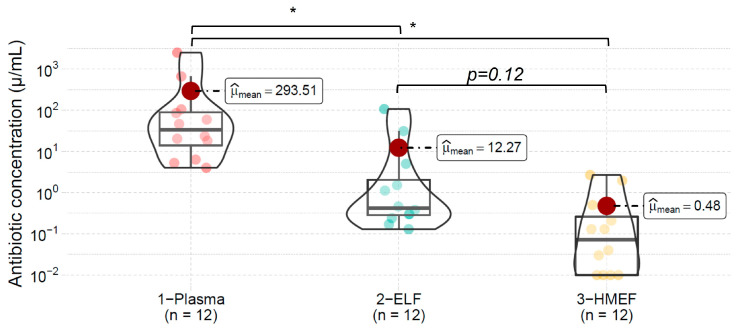
Antibiotic concentration in the three compartments examined. ELF, epithelial lining fluid; HMEF, heat and moisture exchange filter. Symbol * refers to *p* < 0.05 (Bonferroni’s post hoc analysis).

The antibiotic concentrations exhibited significant heterogeneity across the patient population, as depicted in Figure 2. We found no correlation between the concentrations of antibiotics in plasma and ELF (R^2^ = 0.02, *p* = 0.638), plasma and HMEF (R^2^ = 0.02, *p* = 0.62), or between ELF and HMEF (R^2^ = 0.02, *p* = 0.66).

In four HMEF samples, we detected very low antibiotic concentrations. Those samples induced a specific pattern that revealed the presence and identity of the antibiotic used. However, the values obtained were within the lower limit of detection (LOD). To account for this, we arbitrarily assigned a value of 0.01 ng/mL. Conversely, the three control HMEFs showed no signal when testing for PenPcf activity.

## 4. Discussion

In this exploratory study, we identified and quantified the concentration of beta-lactam antibiotics in the exhaled breath condensate collected from the HMEF from critically ill immunosuppressed patients. Moreover, we observed a decremental gradient of antibiotic concentrations from plasma to ELF and to HMEF exhaled breath condensate. However, we did not find any correlation between the three compartments assessed (plasma, ELF, and HMEF exhaled breath condensate).

In previous years, the study of EBC has experienced a notable increase in interest from researchers, primarily driven by the development of novel sampling methodologies and then by advances in analysis techniques. Numerous preclinical and clinical studies have been conducted to assess this matrix exploring various biomarkers in diverse respiratory conditions, including cancer [27], inflammation [28,29], chronic diseases [30], metabolic diseases [31], and other conditions [28]. In addition, the advancement of several technologies such as proteomics [12], metabolomics [30], genomics [32], cellular isolation techniques [33], and the capacity of identification of several volatile markers, such as antibiotics [13], have played a crucial role in advancing research in this field. Recently, Rahimpour et al. employed a copper nanocluster-based sensor to determine the levels of vancomycin in the EBC of five newborns undergoing intravenous treatment with this antibiotic, and they were able to identify antibiotics in all samples and validated their findings by adding a known antibiotic concentration aliquot to the sample. They obtained EBC samples from the waste of the ventilator [34]. Likewise, Khoubnasabjafari et al. conducted a pilot study on healthy volunteers who had received an inhaled monodose of tobramycin. They successfully detected tobramycin in all the EBC samples collected from an expiratory reservoir [35].

McNeil et al. studied EBC from HMEFs from critically ill patients. They suggested that there is a novel, non-invasive method to sample the distal airspace in patients with ARDS accurately. They found a good correlation between proteome isolated from undiluted pulmonary edema fluid and exhaled breath condensate collected from HMEF. In addition, they could discriminate between cardiogenic and inflammatory pulmonary edema based on differences in proteins identified by high-performance liquid chromatography (HPLC) [12]. More recently, Herregodts et al. recognized beta-lactam antibiotics on the exhaled breath condensate collected from spontaneously breathing patients. They used a commercially available device consisting of a mouthpiece with saliva and electrostatic filters to capture bioaerosol particles. They identified antibiotics in all patients (*n* = 9), but similarly to us, they could not find a good correlation between the exhaled antibiotic concentration and the plasma antibiotic concentration [13]. There is no consensus on how to interpret the measures of EBC concentration and how to correlate them with blood-based measurements. Ates et al. describe the temporal evolution of piperacillin/tazobactam levels in EBC and plasma, saliva, and urine samples from a porcine model. Revealing different clearance behaviors in accordance with the different transport mechanisms, using a microfluidic biosensor using penicillin-binding proteins. They also validated their measures at EBC and plasma using HPLC [36]. 

The lack of correlation observed between antibiotic concentrations in different compartments, as reported in our study, can be attributed to various factors and underscores the complex pharmacokinetics of antibiotics within the lungs. Firstly, the plasma levels of beta-lactam antibiotics exhibit significant variability over time, characterized by fluctuating peaks and troughs, and varying ascending or descending slopes, influenced by factors such as clearance and specific drug properties [37]. In our study, plasma samples were only collected at the time of bronchoalveolar lavage (BAL). Consequently, we did not control the time between the antibiotic administration and the plasma sampling, which could lead to discrepancies in the temporal peaks between the examined compartments [36].

In addition, patients had received different antibiotic doses and for different periods when BAL was performed, so some patients were probably in the steady state while others were in the ascendant limb of the plasma time–concentration curve. Finally, the renal clearances and volumes of distribution varied between patients (Table 1). These variables could in part explain the interindividual variability of the ELF/plasma ratio of antibiotic concentration among our patients. Recently, Paal et al. showed a very large interindividual variability from ELF/serum and interstitial fluid/serum of meropenem concentration from lung parenchyma during lung transplantation surgery. In addition, it is important to remark that ELF represents only a regional measure and HMEF-exhaled breath condensate represents a more global lung assessment; this factor also could collaborate with the lack of correlations between ELF and HMEF exhaled breath condensate [38].

ELF antibiotic concentration depends on several factors such as the plasma concentration, protein binding, and physicochemical characteristics of the drug [39]. However, it usually follows a rapid increase after intravenous infusion and then a monoexponential decay. In addition, lung permeability and inflammation may vary regionally through the lungs and among patients [40]. In the same line, the V/Q mismatch may explain different ELF drug concentrations through the lungs, as has been demonstrated with radionuclide tracers and MRI studies [41,42]. Therefore, the lung region chosen to perform the BAL should influence the antibiotic concentration collected, and it may be different throughout different lung regions. However, it is also important to consider the impact of extravascular lung water on the ELF concentration of antibiotics. The presence of increased extravascular lung water or increased capillary leak could lead to the dilution of antibiotics, potentially affecting their concentration in the ELF [43]. 

Antibiotic deposition on the HMEF will result, in part, from an equilibrium between the plasma and ELF compartments. From the ELF, the antibiotic should be transported by tiny drops through airways up to the HMEF [39]; these drops will precipitate in the HMEF depending on physical variables such as temperature gradients, atmospheric pressure, and the composition and size of the HMEF. However, currently, we have no available data on whether HMEFs progressively accumulate antibiotics over time or if it presents a dynamic equilibrium between antibiotic deposition on the filter and/or antibiotic reinhalation. Another point to remark is that many antibiotics can be affected by hydrolysis and photolysis in an aqueous solution, so longer times of permanence on the filter may favor degradation [44]. 

The determination of beta-lactam antibiotics in ELF or HMEF using the PenPcf biosensor has not yet been validated using HPLC. Nevertheless, we previously demonstrated that the concentration of antibiotics in complex matrices such as plasma in the range between 130 and 6500 uM determined by the PenPcf biosensor correlate (R^2^ = 0.99) with concentrations determined by isocratic HPLC [20]. However, the concentration ranges present in ELF and HMEF were one to three orders lower than those of the comparative curve described. Therefore, although the PenPcf biosensor approach has been shown to be accurate and precise in determining antibiotic beta-lactam concentrations in plasma samples, other validation tests oriented to the specific concentration ranges of matrices other than plasma could be performed to confirm its accuracy for other samples.

We observed four HMEF fluid samples in which antibiotic concentrations were above the LLOD (lower limit of detection). However, they were below the lower limit of quantitation (LLOQ), where the amount of antibiotic can be confidently extrapolated. This issue has been extensively studied previously, and it has been shown that excluding the data between the LLOD and the LLOQ increases the bias and decreases the precision of the statistical analysis, especially when the percentage of data discarded is higher than 10%. Although some rules have been described for assigning values to these types of determinations, such as “LLOQ/2”, this rule can only be applied after a well-characterized statistical method [22,23,24,25,26]. In our case, due to the lack of systematic statistical characterization of the method, the concentration value was arbitrarily assigned as the LLOQ value (0.01 ng/mL). In addition, the LLOQ value denoted very low antibiotic concentrations in HMEF samples, which were between 17 to 46 times lower than the corresponding ELF concentration. In our opinion, these values demonstrate that the antibiotic was present in the samples and its concentration was very low.

Despite the advances in the study of EBC, there are scarce data regarding the measurement of antibiotic concentrations in this matrix. We consider this an important issue because one of the main therapeutic objectives in the clinical setting is to achieve proper antibiotic concentrations at the infection site (i.e., lung airspaces), avoiding systemic toxicity and adverse effects secondary to the diagnostic method and to the increasing bacterial resistance secondary to infra-therapeutic levels of antibiotic. To advance towards these objectives requires improving the knowledge of antibiotic pharmacokinetics in different biological compartments of critically ill patients. This information, in the future, could help clinicians to tailor the individual selection, dosage, and administration intervals of specific antibiotics. Thereby, future technological developments could monitor the adequate dosing titration of antibiotics for patients with pneumonia, similar to what happens today with inhaled anesthetics in the operating room [45,46]. 

We acknowledge some limitations in our study: first, we studied a small sample size. Second, we did not use the gold-standard method (HPLC) to measure antibiotic concentration; we consider that the use of HPLC may have been a good tool to measure the very low concentrations of antibiotics as we observed in HMEF exhaled breath condensate. Third, despite the common factor of respiratory failure in our patients, they presented a great heterogeneity in their clinical conditions. Fourth, we did not control the time of sampling. Sixth, we did not perform pharmacokinetic models or repeated measures to understand how the antibiotics were distributed across the different compartments over time. Finally, we cannot discard that the time of permanence of HMEFs could have influenced the antibiotic concentration measured.

## 5. Conclusions

In conclusion, beta-lactam antibiotics can be detected and measured from the exhaled breath condensate accumulated in HMEFs. However, we found no correlations among the plasma, ELF, and HMEF exhaled breath condensates regarding the concentration of antibiotics. Future studies should try to better understand the factors involved in the transport of antibiotics from the alveoli to the HMEF to determine its potential as a novel source for the therapeutic drug monitoring of antibiotics.

## Figures and Tables

**Table 1:** Baseline characteristics of patients.

Patient	BMI	PaO_2_/F_I_O_2_	Antibiotic	Dose (Hours)	Diagnosis	eGFR (mL/min)	Albumin (gr/dL)	HMEF Time (Hours)	SOFA Score	Outcome	Antibiotic Concentration in the Three Compartments Examined. (ng/mL)		
											Plasma	ELF	HMEF
1	21	272	Imipenem	500 mg c/8	Pneumonia	69	2.8	15	9	Alive	104.93	31.07	0.03
2	22.6	216	Imipenem	500 mg c/6	Pneumonia	90	2.9	7	6	Dead	20.28	106.56	0.13
3	30.7	172	Imipenem	1 gr per day	Pneumonia	59	2.6	6	6	Alive	6.32	1.12	2.68
4	39.8	176	Pip/Tazo	4.5 gr c/8	Pneumonia	151	4.2	7	10	Dead	84.90	0.17	0.01
5	16.8	285	Pip/Tazo	4.5 gr c/8	Pneumonia	40	2.2	8	15	Dead	5.26	4.99	1.96
6	17.3	202	Imipenem	500 mg c/12	Pneumonia	17	2.7	11	8	Alive	4.01	0.46	0.01
7	29.1	63	Imipenem	1 gr c/12	ARDS	8	2.4	12	15	Alive	659.51	0.38	0.01
8	27.3	242	Meropenem	2 gr c/8	Pulmonary sepsis	125	2.2	6	6	Alive	46.52	0.13	0.51
9	24	162	Meropenem	2 gr c/8	Pneumonia	126	2.0	6	7	Dead	23.42	0.30	0.01
10	16.8	200	Imipenem	500 mg c/6	Pulmonary sepsis	110	2.3	19	8	Alive	18.18	1.52	0.13
11	26	141	Imipenem	500 mg c/6	ARDS	100	3.3	19	10	Dead	59.22	0.30	0.04
12	30.9	250	Ceftriaxone	2 gr per day	ARDS	11	2.0	12	12	Alive	2489.6	0.24	0.21
Mean	25.1	198				75.5	2.6	10.7	9		293.51	12.27	0.48
SD	6.9	62				49	0.63	5.1	3.2		715	31	0.5

BMI: body mass index; Pip/Tazo: piperacillin/tazobactam; ARDS: acute respiratory distress syndrome; eGFR: estimated glomerular filtration rate; SOFA: sequential organ failure assessment; HMEF time: HMEF hours from HMEF installation; ELF: epithelial lining fluid; HMEF: heat and moisture exchange filter.

## Data Availability

Deidentified data are available upon reasonable request to the corresponding author (J.R.).
